# Four Urgent Actions for the Rights to Culturally Safe Breastfeeding for Aboriginal and Torres Strait Islander Mothers and Babies to Breastfeed in Neonatal Intensive Care Environments

**DOI:** 10.5694/mja2.70237

**Published:** 2026-06-30

**Authors:** Jessica Bennett, Jamie Bryant, Kade Booth, Michelle Kennedy

**Affiliations:** ^1^ School of Medicine and Public Health The University of Newcastle Callaghan New South Wales Australia; ^2^ Neonatal Intensive Care Unit John Hunter Children's Hospital New Lambton Heights New South Wales Australia; ^3^ Hunter Medical and Research Institute New Lambton Heights New South Wales Australia

**Keywords:** breastfeeding, cultural competency, neonatology

## Abstract

Breast milk provides both short‐term and long‐term health benefits and is critical for infants admitted to neonatal intensive care units (NICUs). Presently, there is a large focus on increasing breastfeeding rates among Aboriginal and Torres Strait Islander women; however, culturally safe breastfeeding support is under‐recognised and inadequately addressed in the NICU setting. This perspective highlights cultural and structural barriers and calls for urgent action to include the following four proposed strategies: strengthening the Aboriginal lactation workforce, embedding culturally inclusive education, partnering with community‐controlled services, and developing a culturally specific NICU breastfeeding policy to improve outcomes.

## 1 Introduction

Breastfeeding and the use of human breast milk is globally recognised to provide both short‐term and long‐term health benefits for an infant within the first 1000 days of life [1, 2, 3]. For infants born prematurely or unwell and requiring an admission to the neonatal intensive care unit (NICU), breast milk consumption provides several benefits to their health outcomes. Benefits include immune defence, nutritional benefits, neurodevelopmental outcomes, gastrointestinal development [4] and a reduction in acute health risks such as necrotising enterocolitis and infection risks [5, 6]. The *Australian National Breastfeeding Strategy: 2019 and beyond* provides a national roadmap to increase breastfeeding initiation and duration through supportive breastfeeding‐friendly environments [7]. Aboriginal and Torres Strait Islander peoples were identified as a priority population that requires targeted support to improve current breastfeeding outcomes [7]. Concerningly, to provide culturally appropriate and responsive breastfeeding support as per the strategy, the NICU environment has not been considered as a concern for Aboriginal and Torres Strait Islander families and their infants. Current research indicates that culturally safe care is disrupted in the NICU setting for Aboriginal and Torres Strait Islander families and that there is a lack of culturally safe breastfeeding support available [8]. It is noted in the strategy that the NICU is to be a time of ‘difficult circumstances’ in an infant's breastfeeding journey and that this requires dedicated lactation support and services available [7]. This perspective article builds on the lead author's previously published doctoral research, exploring culturally safe care for Aboriginal and Torres Strait Islander infants and their families in the NICU [8, 9, 10]. Although the work explored parents' experiences of care more broadly, interview data highlighted that cultural breastfeeding support in the NICU is frequently overlooked. This article extends those findings by offering a culturally informed lens to breastfeeding and provides key recommendations to strengthen culturally safe breastfeeding support for Aboriginal and Torres Strait Islander women in the NICU.

The CONSIDER reporting criteria checklist for health research involving Indigenous peoples was completed for this article and can be found in the Supporting Information [11].

## 2 Positionality Statement

To understand the context of this article, I, the lead author, wish to position myself. My name is Jessica Bennett, I am a proud Gamilaroi woman, Neonatal Registered Nurse and postdoctoral researcher. I am a mother of four and two of my children have been admitted to the NICU. My relationality is at the centre of my research. The research team comprises both Aboriginal and non‐Aboriginal researchers, with expertise in Aboriginal and Torres Strait Islander health research. Dr. Kade Booth and Dr. Jamie Byrant are non‐Indigenous postdoctoral researchers with a combined experience of over 20 years in the field. Professor Michelle Kennedy is a proud Wiradjuri woman, social worker, a mother of three and National Health and Medical Research Council Research Fellow at the University of Newcastle with over 20 years' experience working with Aboriginal and Torres Strait Islander communities.

## 3 Barriers to Successful Breastfeeding in the General NICU Population

Parents who experience a neonatal admission with their infant have reported parental stress, trauma and isolation [12, 13]. The nature of the NICU can delay the initiation of breastfeeding and infants can struggle to establish breastfeeding due to gestational age (prematurity), cue‐based feeding, immature growth and development, and neurological maturation (suck, swallow and breathe reflex) [14]. Several studies have established significant barriers to successful breastfeeding in the NICU for the general population. These barriers include insufficient breastfeeding education, breast pump availability, pumping to maintain a breast milk supply or initiation, lack of health provider support and consistency, infant's medical condition and ability to provide skin‐to‐skin care [14, 15, 16].

## 4 Breastfeeding for Aboriginal and Torres Strait Islander Women

Before colonial disruption, breastfeeding was a common practice of feeding Aboriginal and Torres Strait Islander infants [17]. Colonisation destroyed cultural breastfeeding knowledge and enforced non‐Indigenous ways of infant feeding due to the forced removal of Aboriginal and Torres Strait Islander children, compulsory domestic labour, inadequate access to food and water, and unjust government policies that forbade traditional ways of parenting [18]. Successful breastfeeding rates among Aboriginal and Torres Strait Islander women are associated with breastfeeding support that is provided through Aboriginal community‐controlled health services and is underpinned by cultural norms, kinship support, connection to culture and cultural breastfeeding knowledge and support [19, 20, 21]. National data currently indicates that Aboriginal and Torres Strait Islander women have lower breastfeeding rates than non‐Indigenous women, despite no current national data monitoring system and inconsistent reporting and misidentification of Aboriginality in public health settings [18]. This discrepancy fails to effectively inform clinical practice, targeted interventions or support policy development for Aboriginal and Torres Strait Islander breastfeeding practices in the NICU.

## 5 Breastfeeding in the NICU for Aboriginal and Torres Strait Islander Women: Findings From Lead Author's Doctoral Research

Aboriginal and Torres Strait Islander infants experience a higher admission rate to the NICU compared with the general population [9, 22]. During an admission, Aboriginal and Torres Strait Islander mothers demonstrated high intentions to breastfeed; however, a low proportion of infants received breast milk on discharge (86.4% vs. 62.6%) [9]. The number of infants receiving breast milk on discharge from this cohort [9] is below the NSW state average for Aboriginal and Torres Strait Islander women who are partially or exclusively breastfeeding at hospital discharge (72.5%) [23]. In 2024, when analysing the experiences of Aboriginal parents in the NICU, six Aboriginal mothers described breastfeeding as a source of strength to be able to provide for their infants during their NICU admission; however, the nature of the intensive care setting limited cultural practices that upheld a culturally safe breastfeeding environment [8]. We conducted a secondary analysis of these data in October 2025, using thematic analysis to identify themes and recommendations to improve breastfeeding experiences of Aboriginal women in the NICU. This approach centres the voices of the mothers to develop an evidence base to inform meaningful change among a small sample size [24]. Ethics approval was obtained from the Aboriginal Health and Medical Research Council Human Research Ethics Committee (HREC 2080/23), the Hunter New England Local Health District Human Research Ethics Committee (HREC 2023/ETH00910) and the University of Newcastle Human Research Ethics Committee (HREC H‐2023‐0165).

Barriers to a successful breastfeeding journey in the NICU included a low milk supply due to a lack of normal diet and food security while staying in the hospital, parental stress surrounding cultural parenting, maternal health and physical separation from their infant. Inconsistent information and poor communication on breastfeeding a premature infant or expressing breast milk in the NICU also caused mistrust and a lack of opportunity to engage in breastfeeding planning. Some mothers expressed frustrations about their ability to advocate and consent to infant feeding practices, which altered their ability to offer breast milk and devalued their lived experiences and cultural knowledge around breastfeeding practices. ‘I got five sisters that I've seen breastfeed their babies…They put baby in a position, and I know what position my baby's got to be in, how they've got to lay and that’. *(Mother 10—Kamilaroi)*.

Mothers asserted that cultural support is integral to the success of breastfeeding in the NICU. The need for Aboriginal and Torres Strait Islander workforce representation was highlighted and the opportunity to be supported by family and friends, including friendships built between other Aboriginal and Torres Strait Islander NICU parents, was seen to support success towards breastfeeding on discharge. ‘Having friendly parents who, most of them are on their third or fourth baby, and they were just so loving and had so much advice to offer me as a first‐time mom’ *(Mother 13—Wonnarua)*.

## 6 Action Is Needed to Transform Culturally Safe Breastfeeding in the NICU

The NICU is a known barrier to successful breastfeeding practices for Aboriginal and Torres Strait Islander women [19, 25]. These women deserve the right to culturally led and safe breastfeeding support to provide their infant the best possible start to life during their time in the NICU. To drive a culturally informed agenda reform for breastfeeding practices and cultural support in the NICU and neonatal health policy, four clear and distinct recommendations are presented below.

1. Increase the lactation‐trained Aboriginal and Torres Strait Islander NICU health workforce

Priority must be given to increasing the representation of Aboriginal and Torres Strait Islander staff to support culturally safe and culturally led breastfeeding practices in the NICU. This includes the upskilling of the current Aboriginal and Torres Strait Islander neonatal workforce to pursue lactation training and permanent identified positions.

2 Invest in culturally inclusive and appropriate breastfeeding education

Investment is needed in embedding culturally led and co‐designed resources and support into current breastfeeding education and support for Aboriginal and Torres Strait Islander women. This includes providing neonatal‐specific culturally safe breastfeeding education.

3 Community‐grounded and culturally specific breastfeeding support programs

Partnerships should be fostered with Aboriginal community‐controlled health organisations to support public health services to provide culturally safe spaces for Aboriginal and Torres Strait Islander women. This will increase cultural‐led Aboriginal breastfeeding support programs and support the transition back to country with access to community services.

4 Develop culturally specific policy and clinical practice guidelines for culturally safe breastfeeding practices

National culturally specific policies and care guidelines for breastfeeding practices in the NICU must be developed and these should embed Indigenous ways of knowing, being and doing and support cultural parenting considerations for breastfeeding in the NICU, including family support.

## 7 Conclusion

Urgent action is required to offer Aboriginal and Torres Strait Islander women and their babies the appropriate support to empower breastfeeding in NICU environments and beyond. Investment into prioritising the embedding of Indigenous knowledges and leadership, as well as additional, targeted support into the NICU environment is necessary to increase breastfeeding rates and culturally appropriate breastfeeding care to Aboriginal and Torres Strait Islander women and their infants.

## Author Contributions

**Jessica Bennett (Gamilaroi):** conceptualisation, investigation, visualisation, writing (original draft), writing (review and editing). **Jamie Bryant:** conceptualisation, writing (review and editing). **Kade Booth:** conceptualisation, writing (review and editing). **Michelle Kennedy (Wiradjuri):** conceptualisation, writing (review and editing).

## Funding

This project was supported by a University of Newcastle School of Medicine and Public Health Direct Research Costs.

## Disclosure

Not commissioned; externally peer reviewed.

## Conflicts of Interest

Michelle Kennedy is a Guest Editor for the 2026 Special Collection on Indigenous Health and was not involved in any editorial decision‐making about this article.

## Supporting information


**Appendix S1:** CONSIDER statement template.

## Data Availability

In line with Indigenous Data Sovereignty and Aboriginal and Torres Strait Islander Ethical Research Principles, no data sharing is available from this study.
