# Supplementary material for: Four Urgent Actions for the Rights to Culturally Safe Breastfeeding for Aboriginal and Torres Strait Islander Mothers and Babies to Breastfeed in Neonatal Intensive Care Environments
Source: Med J Aust. 2026 Jun 30;224(7):e70237. doi: 10.5694/mja2.70237 (PMC13317532; doi:10.5694/mja2.70237)
Supplement: Supplementary file 1 — Appendix S1: CONSIDER statement template. [file MJA2-224-0-s001.pdf]

MJA
The Medical Journal of Australia

Supporting Information

Supplementary material

This appendix was part of the submitted manuscript and has been peer reviewed.
It is posted as supplied by the authors.

Appendix to: Bennett J, Bryant J, Booth K, Kennedy M. Four urgent actions for the rights to culturally safe breastfeeding for Aboriginal and Torres Strait Islander mothers and babies to breastfeed in neonatal intensive care environments. *Med J Aust* 2026; doi: 10.5694/mja2.70237.

CONSIDER Statement Template

**Governance**
This work has been led by the interest, consultation and needs of Aboriginal and Torres Strait Islander parents in the NICU and has been guided by Aboriginal and Torres Strait Islander voices (Winangali Aboriginal Governance Committee that governed JBe doctoral studies) in partnership with the research team. The Committee influenced the study design, analysis and dissemination of the research within the paper itself.
**Prioritization**
Since closing the gap, Aboriginal and Torres Strait Islander infants are still experiencing admissions to neonatal intensive care units at a rate two times higher than non-Indigenous infants.<br>This research is a priority due to the ongoing the lack of support and literature that is currently available about Aboriginal and Torres Strait Islander health in the NICU in the Australian context and the voices of Aboriginal parents in tertiary NICU experiences of culturally unsafe care for them and their infant.<br>My doctoral research was built from my lived experienced as an Aboriginal woman and neonatal nurse, that then got brought to multiple communities across NSW to understand what communities would like to be researched in this space. The community conversation then built the research work and during qualitative interviews conducted with Aboriginal and Torres Strait Islander mothers, that breastfeeding in the NICU is overlooked. It became apparent that breastfeeding support that is culturally informed and responsive to the mothers' needs is limited in the NICU and requires a call to action to improve current policy and practice. This perspective piece highlights the key actions needed to improve breastfeeding success for Aboriginal and Torres Strait Islander woman in the NICU.
**Relationships (Indigenous stakeholders/participants and Research Team**
The paper has been led by myself, a proud Gamilaroi woman, a Neonatal Registered Nurse and Academic Researcher. During my doctoral research exploring Aboriginal and Torres Strait Islander health in the neonatal setting, breastfeeding was highlighted to be a barrier to receiving culturally safe NICU care. The design, methods, analysis and conduction of the research was influenced by the research team's experiences and knowledges. The team consists of two Aboriginal and Torres Strait Islander researchers with lived experience (JBe, MK) and two researchers with experience in Aboriginal and Torres Strait Islander health research (JBy, KB).
**Methodologies**
Colonisation and contemporary coloniality disrupts cultural caring practices from the forced removal of Aboriginal and Torres Strait Islander children and displacement of peoples from land, country and community. The first authors priority is that the research remains meaningful and impactful for Aboriginal and Torres Strait Islander peoples and the infants in the NICU. Unfortunately, there is minimal literature about Aboriginal and Torres Strait Islander health in the neonatal setting. This perspective highlights the need for culturally safe breastfeeding in the NICU to set the scene for future initiatives, policy reforms and research design. Data is never race-neutral; the design, methodology, and reporting have been influenced by the first author's standpoint and relationality. The research has been Indigenous led, using Indigenous methodologies to ensure that our research is both respectful and promoted culturally appropriate and ethical Aboriginal and Torres Strait Islander health practices. The conduct and reporting of this research adhered to the CONSIDER statement.
**Participation**
This paper reflects on voices of Aboriginal and Torres Strait Islander women and their experiences of breastfeeding in the NICU, individual consent was gained in the Winanga-li study which is reported in another journal. All data was deidentified and stored on a university secured device and only shared with the Aboriginal Governance Committee and research team. In line with Indigenous Data Sovereignty and Aboriginal and Torres Strait Islander Ethical Research Principles, no data sharing is available from this study.
**Capacity**
This research was funded by a University of Newcastle HDR Scholarships for first author JBe, an Aboriginal nurse and researcher, and was based on the interest and collaboration of the authorship team and partnering communities. The collective processes of this research built the capacity of the authorship team.
**Analysis and interpretation**
Secondary data analysis was conducted using NVivo 14 software by the first author, JBe, who conducted a thematic analysis, independently read and re-read interviews to familiarize themselves with the data and coded the stories line-by-line. The data was then presented back to the governance

committee to collaboratively discuss the themes and how it should be best interpretated. This collaborative approach upholds Indigenous research principles and practices by sharing of knowledge and ideas. This ensured that the data was meaningful and could be used to map out what future research and initiatives could occur to support a culturally safe neonatal environment for Aboriginal and Torres Strait Islander families in the NICU environment.
**Dissemination**
The research team have presented these findings back to the governance committee that oversaw JBe doctoral studies. We plan to continue to disseminate these findings beyond this publication. Dissemination will be embedded into future research that the research team conducts in this space, as well as be presented at local and state-based conferences in the neonatal and paediatric settings.
